# Fine Flour Bread

**Published:** 1875-11

**Authors:** 


### FINE FLOUR BREAD.

We speak of the Weak Food which starch affords, in another place, but we seek to impress some of these essential truths upon our readers by repetition. It is not widely known that the snowy flour which is so generally sought after in America, is nearly pure starch. Wheat contains 70 per cent of starch, 15 per cent of gluten, 2 per cent of phosphates, and 13 per cent of water. The gluten and phospates provide the food for the muscles, the brain, and nerves; the starch adds carbon, warmth, and sometimes fat; but the brain and muscular tissue are starved out, and become weak and listless on starch-food. If largely depended upon, white flour, i. e. starch, will surely overheat and excite the system, and will induce inflammations, fevers, neuralgic pains, and many other serious ills. It is not unfair to assert that more of the well-nigh universal nervous diseases from which Americans suffer are induced by white-flour food than by all other influences combined. It is used by all but a few thoughtful people who know how poor a substitute it is for real food.

"Graham-flour," so called, has done a good deal to perpetuate the destructive reign of this wretched starch-monarch. We have all heard of Sylvester Graham's theory that the wheat contains all that is necessary to support vigorous life, and so we buy what some miller tells us is Graham-flour, or what some baker tells us is Graham-bread, little thinking that our "Graham" is only the sweepings of the mill, or the poorest, dirtiest, and least salable stuff, in the possession of the flour-maker or baker.

There never was and there never can be any better food for the average man than can be made from sound, plump, clean, mature, well cultivated, and well ripened wheat. If the miller and the cook would withhold the destructive arts which they employ to destroy the natural appearance of the grain and to disguise its delicate and wholesome taste, it would be the perfect food. The only essential which is demanded in the treatment of wheat-food is that it be LET ALONE. It would be far better that it be eaten raw, than that it be made hot and gritty in grinding, white and starchy by bolting, acrid and unwholesome by the addition of alkalis and acids and saline substances and grease in the cooking processes. As a race we shall be excitable, fretful, nervous, and short-lived, until white flour is banished from our list of foods.

The baker has bleached out his loaves by ammonia and alum, until we have come to suppose that, to be good, a loaf of bread must be as white as the driven snow. There never was a more deplorable fallacy. To be good and wholesome, **bread must NOT be white.** White bread is not totally valueless, but it is approximately so. Bread made from *white* flour is worth, as food, perhaps one tenth as much as bread made from the entire wheat. It is the gray portion of the kernel which contains the brain-food and other muscle-sustaining nutriment, and not the white part.

It is not by any means necessary to all, that wheat should be coarsely ground in order to make substantial and digestible food. In multitudes of cases, it is more grateful to the stomach if ground fine. The stomach and alimentary canal of some people seem to need the active scouring effect of coarse, hully particles, to stimulate them into activity; others are distressed by all coarse or branny foods. Probably the vast majority would feel better and do better on wheat food having in it no large, coarse particles. But there is not one solitary human being alive and in health, who would not feel better, work better, and think better, on bread made of the entire wheat, than on bread from which all the genuine life had been bolted or sifted out. The flour from the "Attrition Mills," so called, proves the best whole wheat product which we have yet tested, and we are inclined to the opinion that it is destined to be universally used by intelligent people to the utter and absolute exclusion, for all purposes, of the white, devitalized flour, now made into worthless, even though pleasant tasting, bread and cake, and pie and pudding. We think the above flour a perfect food: let our friends try it, and tell us if we are right.

